# Mastocytic Enterocolitis: An Overlooked Diagnosis for Unexplained Chronic Diarrhea in a Patient With Colon Polyps and a Family History of Colon Cancer

**DOI:** 10.7759/cureus.37219

**Published:** 2023-04-06

**Authors:** Leeann Hu, Liliana Franco, Jignesh Parikh, Vania Zayat

**Affiliations:** 1 Medical School, University of Central Florida College of Medicine, Orlando, USA; 2 Internal Medicine, University of Central Florida/HCA Healthcare Graduate Medical Education (GME), Orlando, USA; 3 Pathology, Orlando Veterans Affairs Medical Center, Orlando, USA; 4 Pathology, University of Central Florida College of Medicine, Orlando, USA

**Keywords:** intractable diarrhea, mast cell, diarrhea-predominant (ibs-d), systemic mastocytosis, colon polyps, colon cancer, chronic diarrhea, mastocytic enterocolitis

## Abstract

Chronic intractable diarrhea is a common presenting complaint that is often clinically worked up for a wide variety of diseases including inflammatory bowel disease, celiac disease, and hyperthyroidism. When lab results come back normal, patients are often diagnosed with irritable bowel disease-diarrheal subtype, overlooking the potential diagnosis of mastocytic enterocolitis. Mastocytic enterocolitis is an uncommon diagnosis where patients can benefit from mast cell stabilizers that directly target the underlying pathology. Given the broad differential diagnosis of nonspecific diarrhea presentation, a histopathological examination is warranted for definitive diagnosis. We hope to raise awareness of this potentially treatable disease that can be effectively managed with antihistamines. We describe the case of a 63-year-old male patient with a family history significant for colon cancer who presented with intractable diarrhea and was ultimately diagnosed with mastocytic enterocolitis by histopathology. His symptoms were relieved by antihistamine treatment.

## Introduction

Mastocytic enterocolitis presents a clinical picture composed of chronic intractable diarrhea, increased numbers of mast cell aggregates in the mucosal layer of the intestinal wall, and improvement of symptoms with antihistamine or steroidal treatment. The term was first described in the literature by Jakate et al. after observing an increased number of mast cells in the intestinal mucosal biopsies taken from a subset of patients with chronic diarrhea compared to patients with other established causes of chronic diarrhea [1]. They noted that patients with this increase in mast cells in the intestines had improvement of symptoms with antihistamine therapy, sometimes in combination with mast cell mediator release inhibitors. This increase in mast cells has also been observed in patients previously given the diagnosis of diarrhea-predominant irritable bowel syndrome [2,3]. We present a patient with a history of chronic diarrhea and a clinical and pathological picture of mastocytic enterocolitis who has a family history significant for colon cancer. Additionally, we compare the diagnosis of mastocytic enterocolitis to irritable bowel syndrome with diarrhea (IBS-D) and systemic mastocytosis. 

## Case presentation

A 63-year-old man presented to the gastroenterology clinic complaining of intractable chronic diarrhea. He reported three to seven non-bloody loose bowel movements a day for the past four days, associated with urgency. He had been having similar episodes since stopping his medications for mastocytic enterocolitis about one year ago. The patient reported a previous diagnosis of mastocytic enterocolitis that was incidentally found over four years ago but only had colonoscopy evidence from the year prior during which colon biopsies redemonstrated the diagnosis during a period when he had symptoms. The patient was treated at that time with cetirizine and famotidine with resolution of symptoms, but he stopped taking them. The patient stated that cetirizine was given because loratadine gave him headaches when he took it for allergic rhinitis before. He denied nausea, vomiting, melena, hematochezia, fever, chills, and mucus. His medical history also includes hypertension, coronary artery disease, gastroesophageal reflux disease, allergic rhinitis, and migraines. He is a former smoker of 20 years but quit three years ago. His family history is significant for his father and two uncles who had colon cancer. No genetic testing had been performed in the family.

On physical exam, the patient’s abdomen was soft, non-tender, and non-distended, with bowel sounds present and no rebound or guarding. Vital signs were within normal limits. Complete blood count was unremarkable. Since random tryptase was also normal and systemic mastocytosis appeared to be unlikely since his symptoms were limited to the gastrointestinal (GI) tract, an exhaustive systemic mastocytosis work-up was not performed to evaluate for additional criteria.

**Figure 1:**
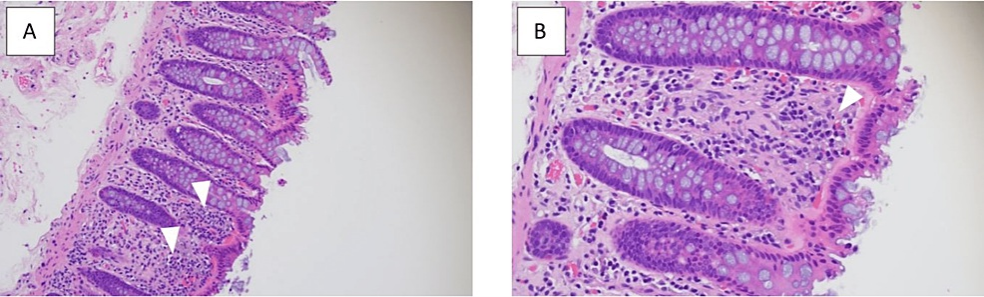
Hematoxylin and eosin stain at 20x (A) and 40x (B) of left colon biopsy showing increased nucleated cells in the lamina propria morphologically suggesting mast cells. No other abnormalities were seen.

**Figure 2:**
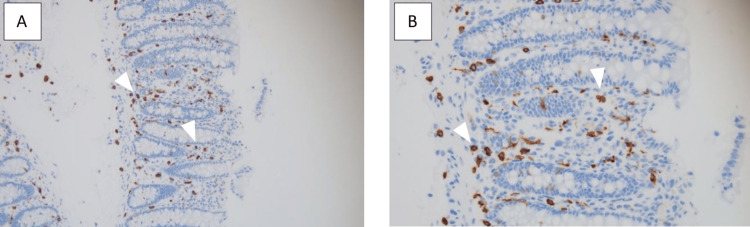
CD117 staining at 20x (A) and 40x (B) highlighting the presence of mast cells within the lamina propria of the left colon biopsy. More than 20 mast cells/hpf can be seen, confirming the diagnosis of mastocytic enterocolitis.

A colonoscopy was performed where one 9 mm sessile tubular adenoma polyp was removed from the cecum and 10 sessile 2 to 5 mm tubular adenoma polyps from the rectum, sigmoid colon, descending colon, and ascending colon. Biopsies taken from the terminal ileum and right colon showed normal architecture. The biopsies of the left colon showed normal colonic crypt architecture but with lamina propria infiltration with oval-shaped cells with central nuclei and dense granular cytoplasm suggestive of mast cells (Figure 1). Upon further characterization with CD117 staining, it highlighted more than 20 mast cells/high power field infiltrating the lamina propria (Figure 2). There was no evidence of active colitis, granulomas, organisms, or chronic crypt destruction on microscopic examination. These results were similar to those from his colonoscopy two years prior.

 

The patient was referred to an allergy clinic and started on 10 mg of cetirizine daily. At the three-month follow-up, he reported resolution of symptoms with cetirizine.

## Discussion

Mast cells have been implicated in an extensive range of mastocytic disorders from systemic mastocytosis to activation secondary to allergic reactions [2,3]. Systemic mastocytosis requires fulfillment of one major criterion and one minor criterion, or three minor criteria. Major criteria are having mast cell aggregates of ≥15 mast cells/hpf in bone marrow or other extracutaneous tissue biopsy. Minor criteria include: 1) ≥ 25% of all mast cells be atypical or spindle-shaped, 2) KIT D816V mutation, 3) expression of CD2 and/or CD25, and 4) baseline serum tryptase level > 20 ng/mL [4]. Given that systemic mastocytosis can affect multiple organ systems, it can have multiple clinical presentations that can mimic many other diseases. Such symptoms include bone pain, abdominal pain, pruritis, neuropsychiatric disturbances, and night sweats [3]. In our patient’s case, he had ≥20 mast cells/high power field in his extracutaneous tissue biopsy that was positive for CD117 (c-Kit). As his symptoms and pathology results show that the mastocytosis is limited to his GI tract, he was diagnosed with mastocytic enterocolitis, the GI subtype of systemic mastocytosis. 

Mastocytic enterocolitis is a rare clinical condition defined by chronic diarrhea in a patient with otherwise normal laboratory findings and histological architecture, except for aggregates of 20 mast cells/hpf or more in the mucosal layer of the intestine [1,5,6]. There is no concrete epidemiology report on how many patients are affected by mastocytic enterocolitis. In a study done with 47 patients with chronic intractable diarrhea with no identified etiology, biopsy specimens were analyzed and 33 were found to have an increase in mast cells of more than 20 mast cells per high-power field [1]. It is believed that the increased local mast cell activity is a more direct cause of the mucosal inflammation, rather than the increased numbers of mast cells alone [2]. This inflammation irritates the enteric nervous system and alters the gut barrier, causing intractable diarrhea [1]. 

Patients typically present with chronic intractable diarrhea without alarm features, similar to diarrhea-predominant irritable bowel syndrome (IBS-D). Our patient did not have the alarming findings of significant unplanned weight loss, melena, history of recent antibiotic use, anemia, or raised inflammatory markers. However, he was found to have tubular adenomatous rectal polyps and a significant family history of colorectal cancer, which warrants closer clinical evaluation. Histological comparison of his colonoscopy results to that of two years prior shows that his descending colon which previously only had mastocytic enterocolitis with no sign of polyps now has tubular adenomatous polyps. Adenomatous polyps are considered a precursor to colorectal cancer; the role of mast cells in predisposition to formation of adenomatous polyps and colorectal cancer through effects on local inflammation has been suggested, and further information is needed to validate this hypothesis [7-9]. Mast cells are seen in polyps in abundant numbers from the onset, and a study conducted on mice has shown that depletion of mast cell numbers can cause remission of existing polyps [10]. Retrospective studies have also found a significant inverse correlation between mast cell density and survival rate from colorectal cancer [11,12]. However, the connection between mastocytic enterocolitis and colorectal cancer still requires further investigation [8,9,13]. This is further complicated by the difficulty in correctly identifying mastocytic enterocolitis as its isolated GI symptoms, nonspecific symptoms, and similarity to IBS-D can cause the diagnosis to be easily missed [14].

Mastocytic enterocolitis and IBS-D patients both typically have normal laboratory findings including erythrocyte sedimentation rate, serum tryptase, and chemical analysis. Tests such as immunoglobulin A tissue transglutaminase, pancreatic elastase, TSH, and fecal calprotectin are often done to rule out other common causes of diarrhea such as celiac disease, hyperthyroidism, and inflammatory bowel disease [15]. Therefore, the distinction between IBS-D and mastocytic enterocolitis relies heavily on the histological identification of mast cells. Immunohistochemical analysis of biopsies with tryptase or CD25 and/or CD117 (c-KIT) staining reveals an increased number of mast cells [16]. CD117 staining is routinely used in our institution for patients whose biopsies have morphological characteristics pointing toward mast cell infiltration which allowed for re-confirmation of our patient’s previous diagnosis of mastocytic enterocolitis. Histological identification of mastocytic enterocolitis therefore can direct treatment goals toward targeting the underlying pathology rather than what would be symptomatic control or medications such as alosetron or eluxadoline in IBS-D.

Successful improvement of mastocytic enterocolitis symptoms can be achieved with the use of antihistamines, differentiating its clinical picture from that of IBS and other causes of chronic diarrhea. Initial treatment consists of histamine H1 and H2 receptor blockers, such as cetirizine and famotidine respectively, that inhibit mast cell release and function. Alternatively, cromolyn sodium can be used to prevent mediator release from mast cells [17]. In patients with refractory symptoms despite the initial treatment, steroids such as oral budesonide and beclomethasone dipropionate have been shown to be beneficial by addressing mucosal inflammation [18]. The presence of high mast cell numbers remaining even after successful treatment has led to the proposed mechanism that the disease is caused by a problem with mast cell activity and its mediators rather than mast cell numbers [2,16,19]. 

The significant improvement of symptoms seen after mast cell-targeted treatment should encourage physicians to consider further pathological evaluation of this condition in patients with unexplained chronic diarrhea with no other clinical or laboratory abnormalities.

## Conclusions

Mastocytic enterocolitis should be considered in patients with unexplainable or intractable diarrhea with normal laboratory findings. Colonoscopy with histological examination might be crucial in identifying mast cell infiltration. Diagnosing this overlooked rare entity leads to proper medical management with antihistamines that are proven to be extremely effective and beneficial, especially compared to symptomatic management. Additionally, we hope that our case can encourage further research into the relationship between mastocytic enterocolitis and colorectal cancer.
